# Semi-analytical finite-element modeling approach for guided wave assessment of mechanical degradation in bones

**DOI:** 10.1080/23335432.2017.1319295

**Published:** 2017-05-06

**Authors:** Dhawal R. Thakare, Alexandre Abid, Daniel Pereira, Julio Fernandes, Pierre Belanger, Prabhu Rajagopal

**Affiliations:** a Department of Mechanical Engineering, Centre for Nondestructive Evaluation, Indian Institute of Technology Madras, Chennai, India; b Department of Mechanical Engineering, Ecole de Technologie Superieure, Montréal, Canada; c Department of Surgery, Centre de recherche l’Hôpital du Sacré-Coeur de Montréal, Montréal, Canada

**Keywords:** Guided ultrasound, cortical bone, SAFE method, soft bone tissue, dispersion curves

## Abstract

Numerical models based on the Semi Analytical Finite-Element method are used to study the characteristics of guided wave modes supported by bone-like multi-layered tubular structures. The method is first validated using previous literature and experimental studies on phantoms mimicking healthy and osteoporotic conditions of cortical bone, and later used to study a trilayer marrow–bone–tissue system at varying mechanical degradation levels. The results show that bone condition strongly affects the modal properties of axially propagating guided waves and indicates that L(0,3) and F(1,6) are suitable modes for assessing the mechanical condition of the bone. The work here reports suitable modal selection and their dispersion properties which would the aid in development of a transduction mechanism for mechanical assessment of bones.

## 1. Introduction

### 1.1. Motivation

Osteoporosis is a serious medical condition characterized by degradation of biomechanical properties of bone, thus increasing fragility and posing an increased risk of fracture. Dual-energy X-ray Absorptiometry (DXA), currently used method for diagnosing osteoporosis, can be used to estimate the bone mineral density (BMD) by gaging the absorption level of the X-ray beams by the bone (excluding the soft tissue absorption). However, this method is intrinsically insensitive to mechanical properties or the micro-architecture of the bone (Kanis et al. 1994). DXA may also provide skewed results as a result of indirectly calculating fat mass (Minati 2014), and is not optimal for detection of high fracture risk (Kanis 1994; European Community 1998). Being a 2D projectional measurement, areal BMD is, for instance, also unable to distinguish between differential changes occurring in the cortical and trabecular bones at the femoral neck (Grimal et al. 2013). Other methods such as X-ray Quantitative Computed Tomography allow for much higher resolutions but are limited to certain skeletal sites and involve processing and technical challenges (Adams 2009; Cheung et al. 2013).

Guided ultrasonic waves have been studied as a potentially convenient, inexpensive, and radiation-free alternative method for characterizing cortical bones (see e.g. Tatarinov et al. 2005; Moilanen et al. 2008). For osteoporosis detection, guided waves could potentially characterize the bone as it is sensitive to change in elastic properties of the cortical and trabecular bones as well as to the presence of bone structural features. They may also allow for independent analysis on the cortical and trabecular compartments of the bone to characterize them separately and thus the results can be combined to assess the fracture risk for the complete bone system (Laugier & Haïat 2011).

Set in this context, this paper seeks to study the dispersion characteristics of low-frequency-guided wave modes in the multi-layered bone system with soft tissue and marrow. The studies are carried out using an implementation of the semi-analytical finite-element (SAFE) method validated against literature and experiments with bilayer bone-mimicking phantom tubes. The SAFE method involves a Finite-Element (FE) representation of only the cross-section of the waveguide as it assumes that the wave field is harmonic in the wave propagation direction (x_3_ axis in this paper). The governing wave equations are expressed in the form of an Eigen value problem where each Eigen value represents an associated wave number. The SAFE approach has been detailed further in Section 2.2.

Following the computation of the dispersion properties of the bone system, the paper then attempts to identify guided wave modes which are most suitable for the assessment of mechanical degradation in bones. This is based upon the velocity difference shown by the guided wave modes between healthy and multiple degraded conditions. This then would then serve as a theoretical framework which can be utilized to design a transduction system for characterizing the bone condition. Hence suitability of guided wave modes in a practical implementation has also been discussed.

### 1.2. Background

Computational and experimental studies of Lamb waves in bone-mimicking plates incorporating viscoelasticity, anisotropy, and soft tissues have been reported in recent years (see: Naili et al. 2010; Nguyen and Naili (2012), (2013); Chen et al. 2012; Foiret et al. 2014; Bochud et al. 2015). More recently, modal characteristics in tubular waveguides (Bossy et al. 2004) have been studied, with reports on how features such as fracture-healing (Protopappas et al. 2006) and tube geometry, viscoelasticity and anisotropy of the bone, marrow, and surrounding tissue affect wave propagation (Haïat et al. 2011; Moilanen et al. (2007), (2008); Ta et al. 2009; Chen & Su 2014; Lee & Yoon 2012). However, most previous Lamb-based and 3D tubular (cylindrical)-guided wave studies do not address the essential interactions between the bone, tissue, and marrow and its influences on guided wave propagation.

In the work reported here, an attempt is made to predict the dispersion curves for axially propagating guided waves in the bone system using the SAFE method (explained in Section 2.2. of this paper). The SAFE approach allows incorporation of features such as bone anisotropy, surrounding tissue layers, and the viscoelastic marrow regime without any major changes in its central formulation. Moreover, irregular cross-sections can also be considered, as long as these are axially uniform.

## 2. Methods

### 2.1. Problem studied

#### 2.1.1. Validation models

**Figure 1.**
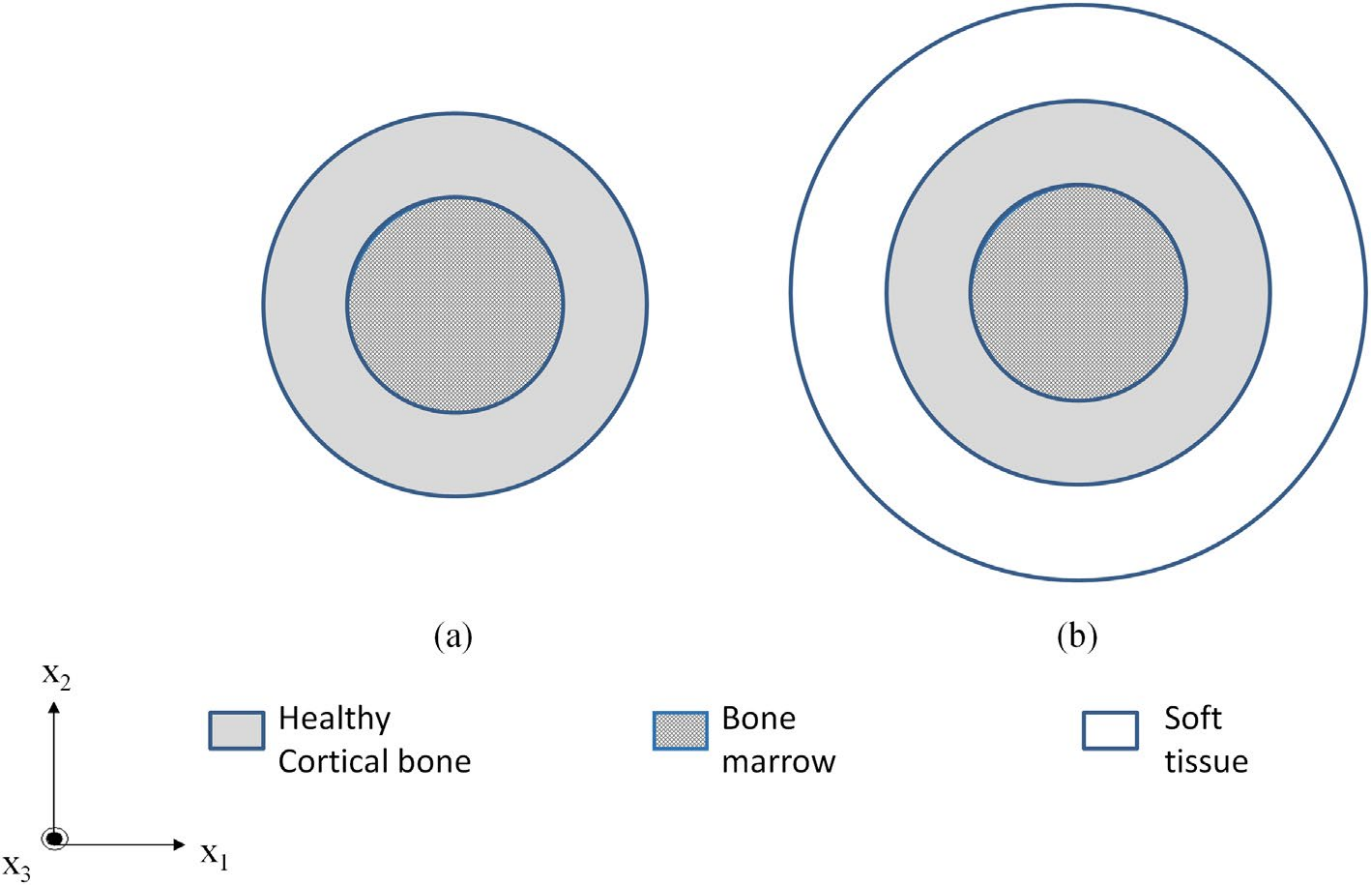
Illustration of (a) model 1 – Cortical bone (*inner radius 7 mm and thickness 3 mm*) filled with marrow and (b) model 3 – Cortical bone additionally surrounded by tissue (*thickness 5 mm*).

We first validated our SAFE implementation for modeling bone systems against results obtained from a previously published paper by Chen and Su (2014). The material properties for this study (called model 1) are shown in Table 1. Following this, we also compared our SAFE modeling approach against experimental studies performed on bone-mimicking phantoms in a healthy and osteoporotic state (called model 2). The cross-sectional view of these phantoms is shown in Figure 1(a) which involves a healthy/osteoporotic cortical bone (inner radius 7 mm and thickness 3 mm) filled with a viscous marrow. For the purpose of SAFE simulations, all materials were assumed as isotropic. The marrow was modeled as an equivalent visco-elastic isotropic solid with complex material constants whose imaginary part accounts for the damping effect (Fan 2010). The properties for model 2 are tabulated in Table 2.

**Table 1.** Material properties for model 1 (validation model of cortical bone coated with/without fluid layer).

Material	Young’s modulus (GPa)	Poisson’s ratio	Density (kg/m^3^)	Bulk’s modulus (GPa)
Cortical bone	16.46	0.373	1850	–
Fluid layer	–	–	1000	2.2

**Table 2.** Material properties for cortical bone and tissue Wydra and Maev (2013) used in the bone models (Bone models 2&3).

Material	Young’s modulus (GPa)	Poisson’s ratio	Density (kg/m^3^)
Healthy cortex	14.95	0.31	2300
Osteoporotic cortex	12.17	0.31	2150
Soft tissue	6	0.43	1250

#### 2.1.2. Bone model

Finally, we consider a multiple layered cortical bone model, (model 3) which is based on the properties of the bone phantoms but bear an extra outer coating layer of soft tissue. Figure 1(b) illustrates model three involving a cortical bone (inner radius 7 mm and a thickness of 3 mm) filled with viscous marrow and coated with soft tissue (thickness 5 mm). Model 3 will be studied at varying levels of degradation, where the Young’s modulus and density of the healthy cortical bone is reduced in steps and dispersion characteristics for each level are compared against the healthy case. Properties for varying degradation levels and soft tissue are shown in Tables 2 and 3, respectively.

**Table 3.** Material properties for varying levels of degradation w.r.t healthy cortical bone in model 3-cortical bone (inner radius of 7 mm & thickness 3 mm) filled with marrow and coated with tissue (5 mm).

Osteoporosis level	Young’s Modulus (GPa) (%age reduction)	Poisson’s ratio	Density (kg/m^3^) (%age reduction)
1	11.96 (20%)	0.31	2145 (7%)
2	10.465 (30%)	0.31	2070 (10%)
3	8.97 (40%)	0.31	1993 (13%)

### 2.2. SAFE method implementation

In this paper, we use the SAFE approach presented by Predoi et al. (2007) and Castaings and Lowe (2008). The procedure, given in some detail in our prior publications in the general context of guided ultrasound in complex media (see Pattanayak et al. 2015; Ramdhas et al. 2015; Manogharan et al. 2016), is briefly outlined below.

Equation (1) expresses the equilibrium wave equation as a 2-D Eigen value problem to be solved for wave number *k* in the propagation direction:

(1) $$\;\;\;\;\;C_{prqs}\frac{\partial^{2}U_{q}}{\partial x_{r}\partial x_{s}}+I\left(C_{p3qr}+C_{prq3}\right)\frac{\partial (kU_{q})}{\partial x_{r}}-kC_{p3q3}\left(kU_{q}\right)+\rho \omega^{2}\delta_{pq}U_{q}=0\;\;\;\;\;\;\;\;\;\;\;\;\;\;\;\;\;\;\;\;\;$$

where the subscript $q\in \left\{1,2,3\right\}\;\text{and}\;r,s\in \left\{1,2\right\}$, *U* is the displacement, *ω* is the angular frequency and the coefficients *C*_*prqs*_ are stiffness moduli which depend on the type of material, and *δ*_*pq*_ is the Kronecker delta symbol.

In our implementation, Equation (1) is solved using the Eigen value formulation available in a commercial FE package (COMSOL Multiphysics® users guide, Version 3.2a), in the general form:

(2) $$\nabla \;.\left(c\nabla U+\alpha U-\gamma \right)-\beta \nabla U-aU+\lambda d_{a}U=0$$

where the coefficients *c*, *α,* and *β* depend on the material stiffness properties, *a* is a function of mass density and angular frequency, *d*_*a*_ depends on stiffness properties, mass density, and angular frequency and γ,λ are null in our case.

All matrix coefficients used in Equation (2) are given by Predoi et al. (2007). For each frequency considered, the wave-number k was obtained using this analysis. The actual possible modes are obtained based on the higher axial power flow in the bone, which represents the propagating mode guided along the bone. The phase velocity *V*_*ph*_ = *ω/k* is then calculated for all propagating modes.

### 2.3. Experimental studies with bone phantoms

Experimental studies were performed on bone phantoms to validate the SAFE approach. Phantoms of cortical bone were acquired from the Institute for Diagnostic Imaging Research, University of Windsor, Ontario, Canada (see Wydra & Maev 2013). The dimensions were chosen to be similar to the middle section of a typical cortical bone like the radius. The phantoms were made of a specially designed composite material using epoxy resin and alumina powder (Wydra & Maev 2013) which closely mimics the cortical bone with properties as shown in Table 2. The phantoms were 150-mm long filled with a polyurethane material which matches the acoustical properties of bone marrow (Bulk modulus 2.2 GPa, viscosity of 37cP and a density of 1000 kg/m^3^) (Gurkan & Akkus 2008).

**Figure 2.**
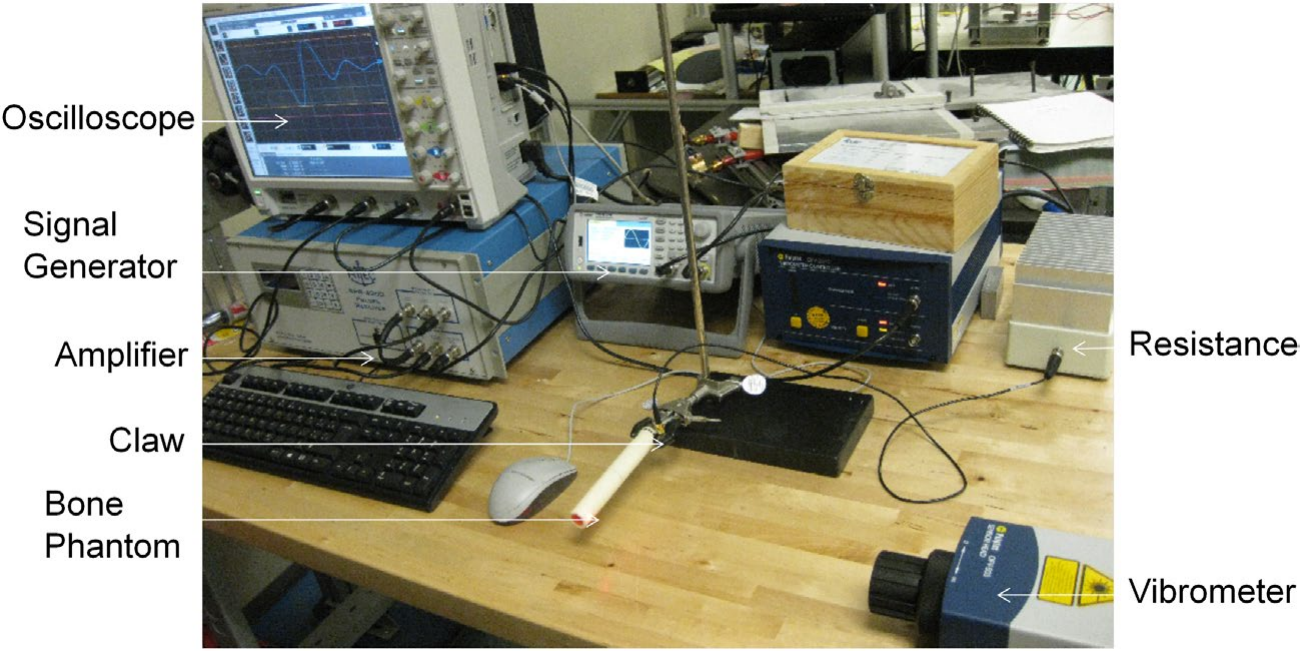
A photograph of the experimental setup used for experimental validation studies.

The experimental setup, as shown in Figure 2, comprised of ultrasonic transducer, either Shear (for instance, Olympus V125-RM, central frequency of 2.25 MHz and diameter of 6.35 mm) or Longitudinal (for instance, Olympus V154-RM, central frequency of 2.25 MHz and diameter of 12.7 mm) and a laser Doppler vibrometer (Polytec OFV-2570). In order to scan a range of frequencies, the input signal was set to a five cycle Hanning windowed toneburst with a center frequency between 50 and 200 kHz as appropriate. The peak voltage used in all measurements was 200 V. The laser vibrometer picked up the stress waves at the receiving end and hence this allowed us to capture the transmitted signal amplitude vs. time. The experimental velocities were then extracted using the Short-Time Fourier Transform (STFT) (Kwun et al. 1999; Niethammer et al. 2001; Ta et al. 2006). The STFT was used to provide a time-amplitude representation as a function of frequency. The time-of-flight of each mode was then extracted as a function of frequency.

The material and geometrical properties of the phantoms were adapted into model 2 which involves a healthy/osteoporotic cortical bone filled with a viscous marrow. The cortical bone has been modeled as an isotropic solid with properties as given in Table 2 and the bone marrow has been modeled as an equivalent viscoelastic solid admitting complex modulus (see e.g. Fan 2010).

## 3. Results

### 3.1. Validation against previous literature

**Figure 3.**
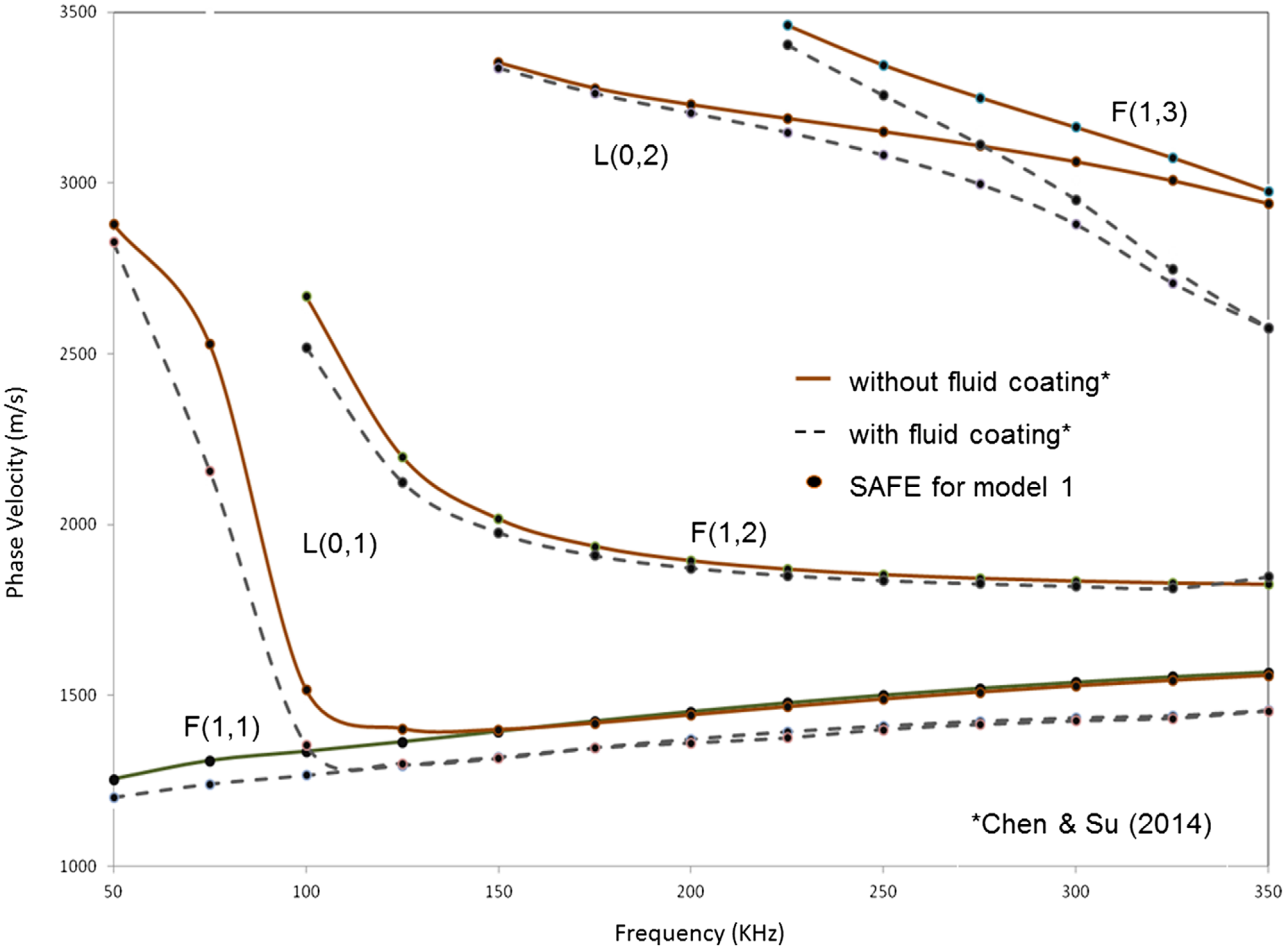
Phase velocity dispersion curves from SAFE analysis (dots) and from Chen and Su (2014) (solid line, dashed line), for model 1 (cortical tube coated without and with a fluid layer, respectively).

The results obtained from SAFE analysis and the published results for model 1 involving a cortical tube with and without a fluid coating are shown in Figure 3. The dots represent the results obtained using the SAFE approach presented in this paper, for both the above cases. As seen from the figure, the results show an excellent match with the theoretical dispersion curves published in the above paper with a maximum error of 2% between the computed and reported velocities.

### 3.2. Experimental validation

**Figure 4.**
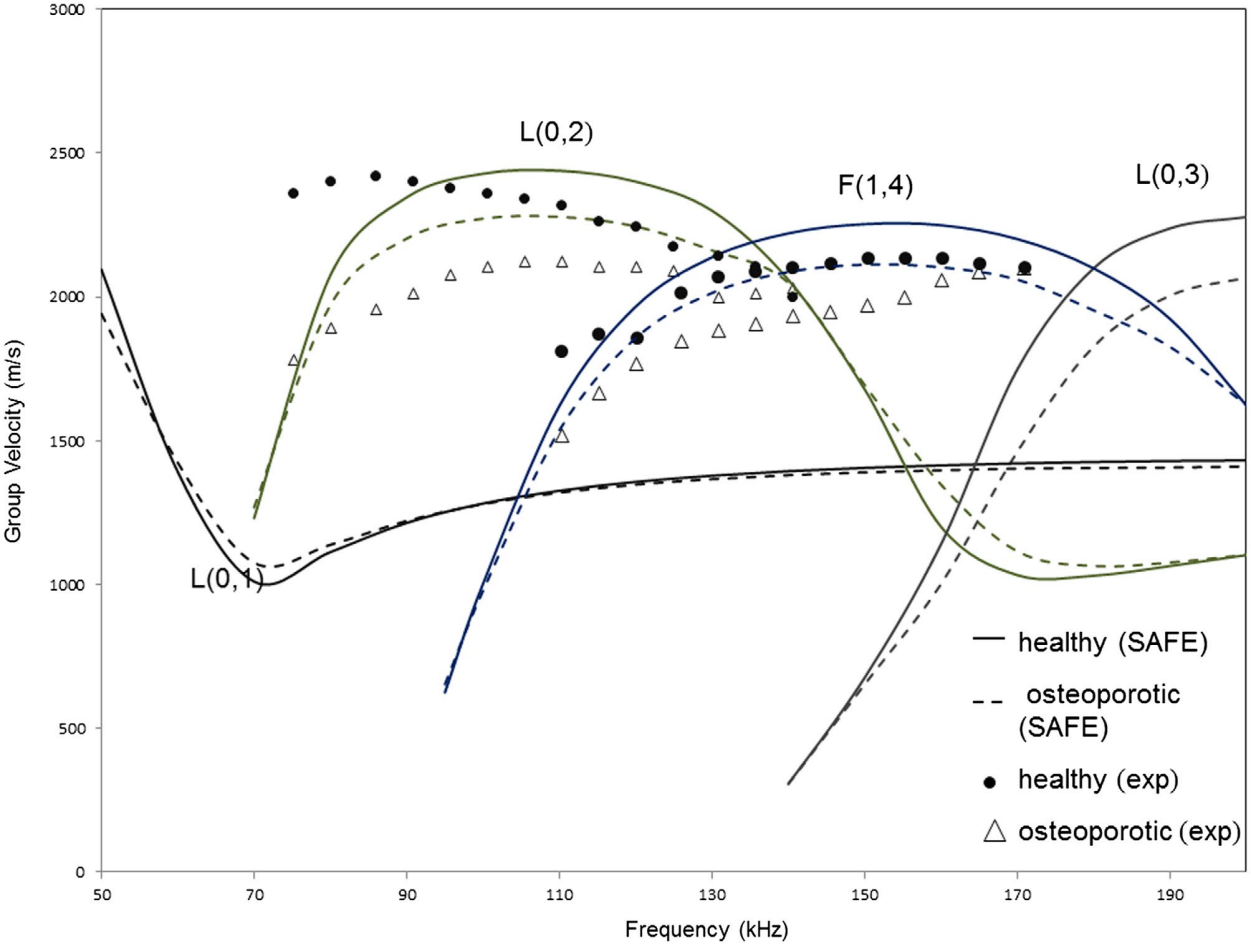
Group velocity dispersion curves (for longitudinal modes) from SAFE analysis (solid – healthy cortex filled with marrow, dashed – osteoporotic cortex filled with marrow) and from experiments (marked) for model 1.

The group velocity dispersion curves have been obtained using SAFE for model 2, as the group velocity can be easily determined using experimental procedures. Results obtained from SAFE to experiments are compared in Figure 4, for a frequency range of 50–200 kHz, in order to limit the number of modes existent in the range and thus simplify the experimental post-processing procedure.

Overall, the experimental results are within 5% of those predicted by SAFE simulations, and this gives confidence to our simulation approach. The manufacturing process of the phantom has about 2% variability in material parameters (Wydra & Maev 2013).

### 3.3. Dispersion characteristics for cortical bone coated with soft tissue

**Figure 5.**
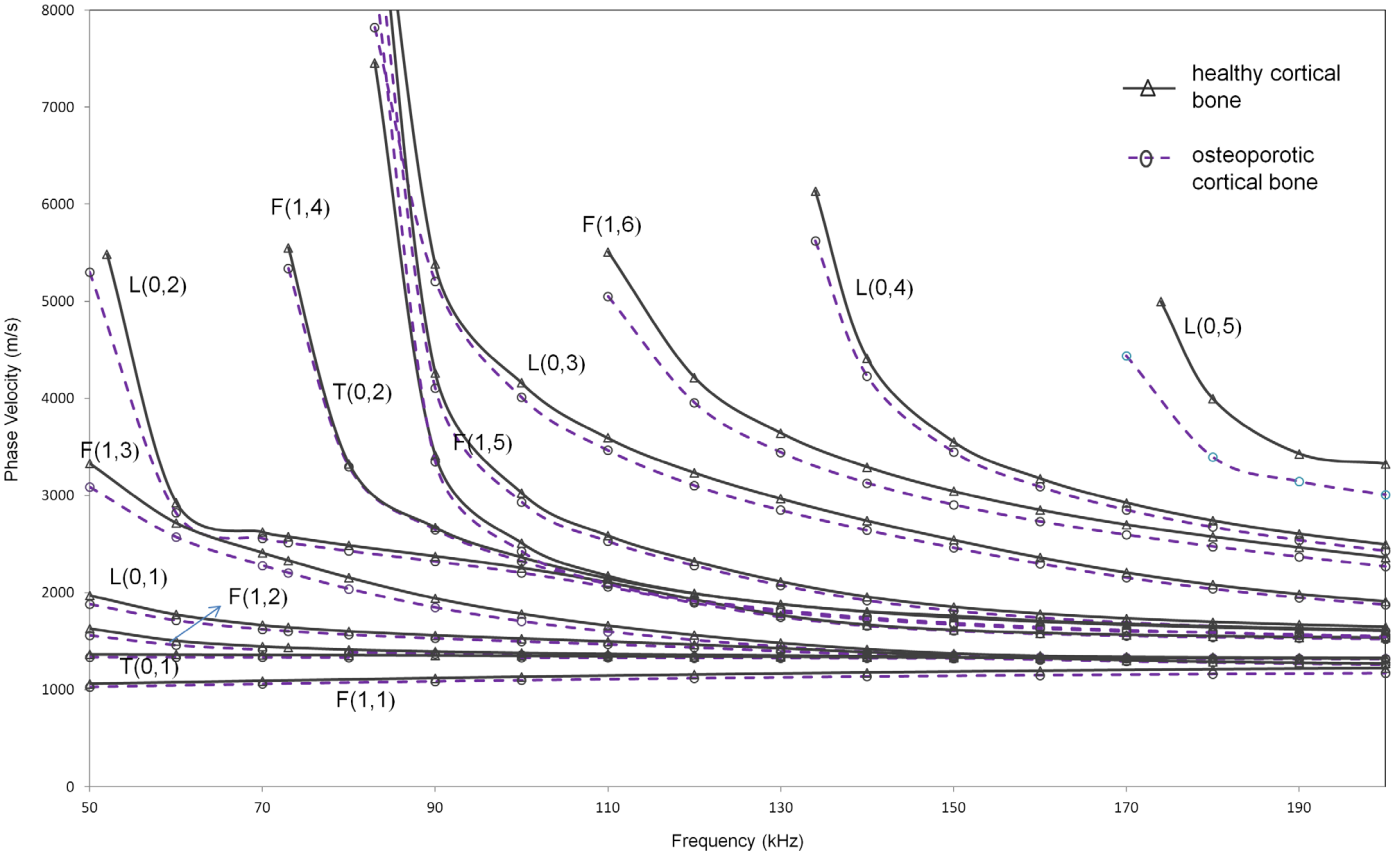
Phase velocity dispersion curves from SAFE analysis for extended model 2 (solid, dashed) (healthy and osteoporotic cortex filled with marrow and coated with soft tissue).

The method was then extended to a more realistic bone structure such that it includes a coating of soft tissue on the outside, in addition to marrow on the inside. Phase velocity dispersion curves obtained using SAFE for modified model 2 with tissue coating are shown in Figure 5. As observed from the figure, the dispersion characteristics in case of soft tissue have the cut-off frequencies of the various modes reduced and hence many modes are now present in the same frequency range.

From a practical consideration, longitudinal modes are preferred for structural assessment, since longitudinal modes are axisymmetric and hence possess a simpler mode-shape. They are also generally less dispersive, have low cut-off frequencies, are easier to generate and receive and hence more conveniently implementable in a practical scenario. However, since large out-of-plane displacement is convenient from a transducer location over a subject’s skin in the final practical case, the suitability of both axisymmetric and non-axisymmetric modes is investigated here.

The most promising longitudinal mode that has a notable velocity difference in this case is the L(0,3) mode with an average velocity difference of approximately 142 m/s (~3.7% reduction) in the frequency range of 90–130 kHz. Among non-axisymmetric modes, F(1,6) mode seems suitable as it shows the highest average velocity difference of 156 m/s (~4.77% reduction) in the frequency region of 120–180 kHz. The numerical comparison of L(0,3) and F(1,6) modes is shown in Table 4. This case will be discussed further in Section 4.1 which investigates the effect of varying levels of degradation.

**Table 4.** Numerical comparison of the phase velocity of suitable modes from SAFE for extended model 2 (healthy and osteoporotic cortical bone filled with marrow and coated with tissue).

Frequency (kHz)	Mode	Phase velocity (m/s)		Phase velocity difference
		Healthy	Osteo-bone	
90	L(0,3)	5386.60	5208.45	178.15
100		4164.35	4011.04	153.31
110		3593.24	3466.67	126.57
120		3237.17	3104.52	132.65
130		2969.57	2850.65	118.92
Average velocity difference				141.92
120	F(1,6)	4216.58	3955.12	261.46
140		3293.90	3128.70	165.20
160		2853.04	2732.92	120.12
180		2576.12	2475.02	101.09
Average velocity difference				156.34

## 4. Discussion

### 4.1 Effects of bone anisotropy and porosity

Although a bone is anisotropic and poroelastic, it has been approximated as an isotropic material in the literature (Nicholson et al. 2002; Protopappas et al. 2006; Moilanen et al. 2007; Ta et al. 2009; Chen & Su 2014). Cortical bone is a compact material with a small degree of internal porosity, which varies between 0 and 10% in healthy and young subjects and may increase up to 10–18% in disease states (Cooper et al. 2004; Nishiyama et al. 2010) depending upon the measurement site. However, with the use of guided waves we are looking at wavelength of modes in the order of few millimeters, which is much larger than the porosity in a typical cortical bone. This serves as a basis for our assumption of homogeneity of the cortical bone.

### 4.2. Varying levels of mechanical degradation

Based on properties of the bone phantom used in the experimental study, our results showed that the guided wave phase velocity reduces in a degraded bone as compared to a healthy bone. In order to observe if this is a general and consistent trend, further levels of material property degradation were considered. Let us examine the lowest longitudinal mode L(0,1). Since it is a dispersive mode, we will look at the low-frequency asymptote for L(0,1) mode which is given by:

(3) $$V_{p}=\;\sqrt{K\left(\upsilon \right)\frac{E}{\rho }}$$

where *E* is the Young’s modulus, *υ* is the Poisson’s ratio, *K*(*υ*) is a function of Poisson’s ratio, and ρis the density. Since the Poisson’s ratio is constant, it follows from Equation (3),

(4) $$\frac{\Delta V_{p}}{V_{p}}=\frac{1}{2}\left(\frac{\Delta E}{E}-\frac{\Delta \rho }{\rho }\right)$$

If the ratio of relative reduction in modulus to relative reduction in density (i.e. $\frac{\Delta E}{E}/\frac{\Delta \rho }{\rho }$) is kept constant, then we can see from Equation (4) that the velocity would decrease with increasing level of degradation (i.e. decrease of both the modulus and density). This ratio has been chosen as approximately 3 in adherence to the properties of the bone-mimicking phantoms used. .In order to investigate this trend, the following section discusses results with three additional levels of degradation on how this affects the phase velocity and the suitability of the propagating modes.

**Figure 6.**
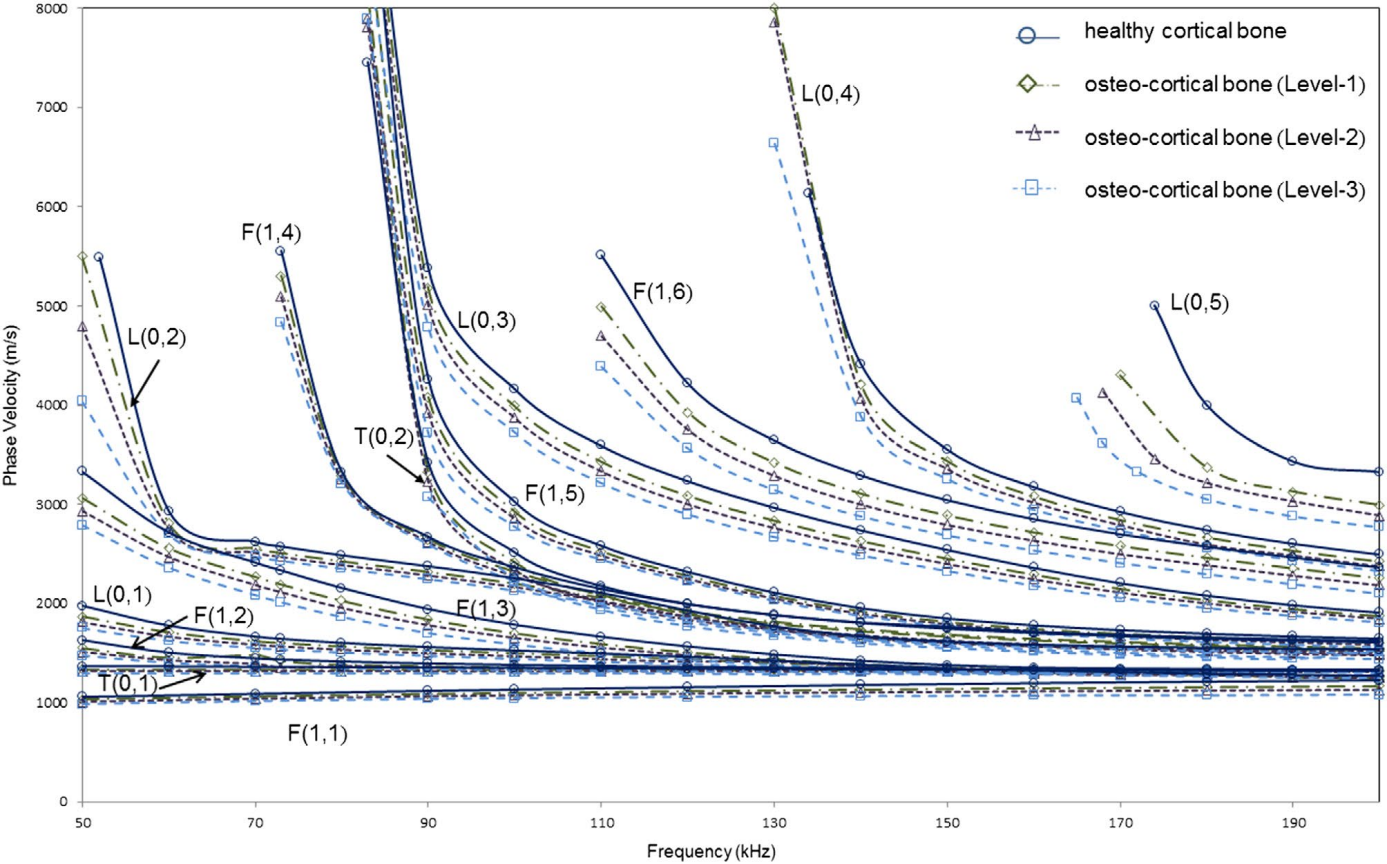
Phase velocity dispersion curves for suitable modes from SAFE analysis for varying osteoporosis levels (Levels 1, 2, and 3) of model 3 (healthy cortex filled with marrow and coated with soft tissue).

We now consider model 3, where three levels of degradation have been considered with material properties as shown in Table 3. A higher degradation level corresponds to a greater decrease in the material properties and therefore models a more degraded bone structure. However as explained before, the degradation levels have been selected such that the ratio of relative reduction in Young’s modulus to relative reduction in density ($\frac{\Delta E}{E}/\frac{\Delta \rho }{\rho }$) is maintained constant. Guided wave phase velocity dispersion curve for model 3obtained using SAFE is presented in Figure 6. It shows that higher the degradation of the bone, lesser the phase velocity of any guided wave mode. This is evident as at a particular frequency, the phase velocity of any mode at that frequency decreases as the degradation level increases.

**Figure 7.**
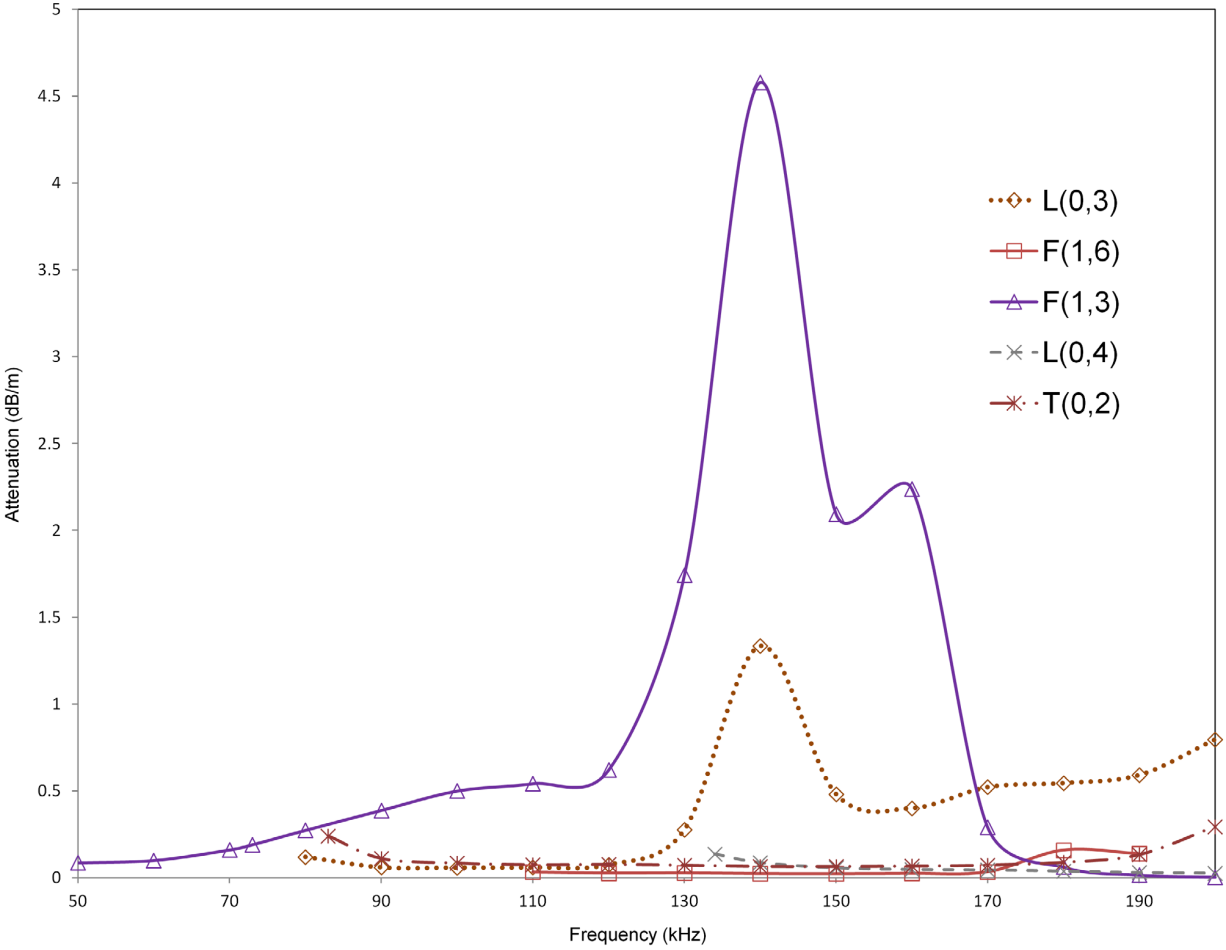
Attenuation dispersion curves for suitable modes from SAFE analysis for varying osteoporosis levels (Levels 1, 2, and 3) of model 3 (healthy cortex filled with marrow and coated with soft tissue).

As seen from Figure 6 and the numerical comparison of the modes as shown in Table 5, F(1,6) and L(0,3) show the highest velocity difference in all the three cases. In the frequency range of 100–140 kHz, L(0,3) shows an average phase velocity difference (with respect to healthy case) of 145 m/s (~4.33% reduction) for Level-1, 236 m/s (~7.1% reduction) for Level-2, and 346 m/s (~10.3% reduction) for Level-3. In the frequency range of 120–200 kHz, F(1,6) shows an average phase velocity difference (with respect to healthy case) of 159 m/s (~5.13% reduction) for Level-1, 258 m/s (~8.35% reduction) for Level-2, and 373 m/s (~12.1% reduction) for Level-3. This is further supported by the relative attenuation of these modes in the case of the healthy cortical bone as shown in Figure 7. These results agree with the findings of modified model 2 (with soft tissue coating) to confirm that F(1,6) and L(0,3) are suitable modes for assessing bone condition in the presence of soft tissue.

**Table 5.** Numerical comparison of the phase velocity of suitable modes from SAFE for varying levels of degradation for model 3 (cortical bone filled with marrow and coated with tissue).

Frequency (kHz)	Mode	Phase velocity from SAFE				Difference between healthy and		
		Healthy	Osteo-Level-1	Osteo-Level-2	Osteo-Level-3	Level-1	Level-2	Level-3
100	L(0,3)	4164.35	3988.90	3868.66	3721.04	175.45	295.68	443.31
110		3593.24	3433.33	3331.60	3211.56	159.91	261.64	381.68
120		3237.17	3089.25	2999.25	2895.18	147.92	237.92	341.99
130		2969.57	2837.77	2758.56	2666.32	131.80	211.00	303.25
140		2742.40	2632.45	2564.00	2481.31	109.96	178.40	261.09
Average phase-velocity difference						145.01	236.93	346.26
120	F(1,6)	4216.58	3923.00	3751.49	3561.22	293.58	465.09	655.36
140		3293.90	3109.81	3001.43	2877.98	184.08	292.47	415.92
160		2853.04	2719.45	2635.75	2535.10	133.58	217.29	317.94
180		2576.12	2463.72	2388.49	2295.69	112.39	187.63	280.42
200		2364.78	2257.30	2185.32	2098.44	107.48	179.46	266.34
Average phase-velocity difference						159.75	258.79	373.45

### 4.3. Power flow and mode excitability

**Figure 8.**
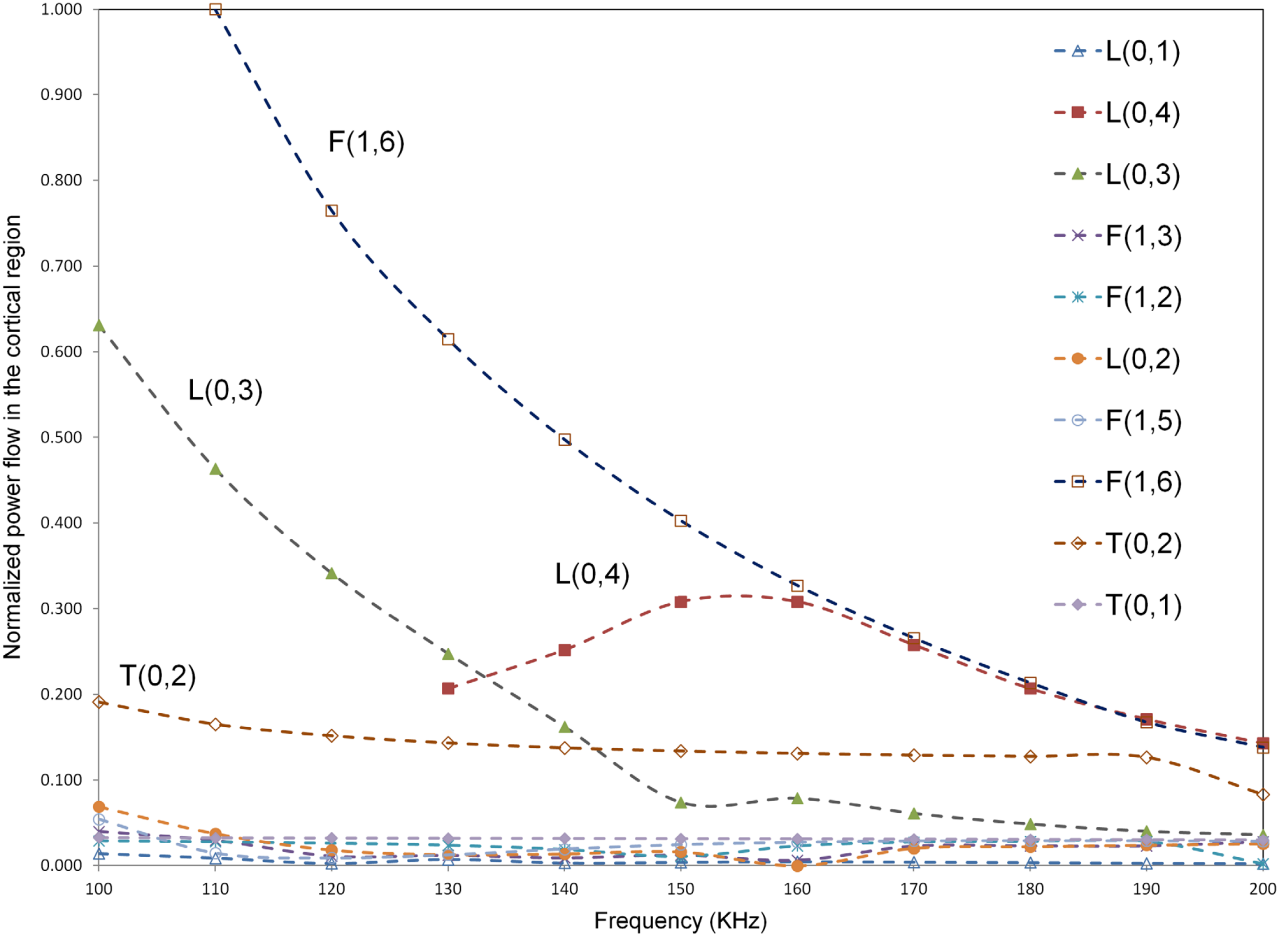
Variation of normalized power flow in cortical region with frequency for model 3 at level 3 osteoporosis (40% reduction in modulus and 13% reduction in density).

In order to illustrate the relative energy in the cortex region of the bone across different guided wave modes, the power flow in the axial or propagating direction (Castaings & Lowe 2008) (*z*-axis) in the cortex compartment alone was calculated for all modes using SAFE in the frequency region of 100–200 KHz. The power flow was calculated using the Poynting vector which can be quantified as follows

(5) $$P_{x_{3}}=-\text{Re}[\left(\frac{I\omega }{2}\right)\left(u_{1}^{*}\sigma_{31}+u_{2}^{*}\sigma_{32}+u_{3}^{*}\sigma_{33}\right)]$$

where $\sigma_{31},\sigma_{32}$ and $\sigma_{33}$ are the corresponding axial stress components, $u_{1}^{*}u_{2}^{*}$ and $u_{3}^{*}$ are the complex conjugate of the vertical, horizontal, and axial displacements. The bone model used for illustration was model three at level 3 degradation. The power flow was then normalized with respect to the highest power flow observed across all modes at a particular frequency, which was F(1,6) mode in this case, at 110 KHz. The variation of normalized power flow in cortex region with frequency is shown in Figure 8. As seen from the figure, F(1,6) mode shows highest power flow in the cortex compared to all other modes. Additionally, L(0,3) mode shows high-power flow after F(1,6) up to a frequency of 130 KHz and previously L(0,3) mode was identified to be a promising mode in a frequency regime of 100–130 KHz. Hence, the above power flow analysis confirms that F(1,6) and L(0,3) are suitable candidates for bone characterization, since across all modes these modes show a relatively high concentration of energy in the cortex region.

### 4.4. Modeling assumptions

The work reported here assumes the bone to be uniform in the axial direction and having a circular cross-section with isotropic properties. The properties of the cortex were decided based upon the phantoms used for experimental studies, while the soft tissue properties were chosen based on previously published studies. Any departure from the above assumptions might influence the absolute values of the velocities reported for the guided wave modes supported by the bone system. However, the study aims to show that given a bone system incorporating a soft tissue and marrow, the modes F(1,6) and L(0,3) would potentially show higher velocity differences between healthy and degraded conditions. For instance, a study investigating the effect of dimensional degradation (change in thickness) in an anisotropic bone system shows similar results as the work reported here (Thakare et al. 2016). The non-uniformity in the bone structure in the axial and radial directions can also affect the dispersion characteristics of the bone system and hence needs further consideration.

## 5. Conclusions and further work

The paper presented a SAFE approach for analyzing guided wave dispersion behavior toward bone condition assessment and detection of osteoporosis. Experimental and SAFE results show a similar trend in the behavior of the dispersion characteristics however exhibiting a noted and measurable offset in velocity values. SAFE simulations of a bone model involving different levels of degradation and a coating of soft tissue show that L(0,3) and F(1,6) have the highest velocity difference in the studied frequency range.

This work is only the initial step of modal selection for guided wave assessment of bone condition. Once the optimal mode has been identified, suitable transducers capable of practical inspection will have to be developed. The transducer development process will rely on excitability of the mode (see for example, surface displacement/axial power flow definition in Wilcox et al. 2001) which can be extracted from SAFE models as described in the present paper.

## Disclosure statement

No potential conflict of interest was reported by the authors.

## Funding

This work was supported by the Shastri Indo-Canadian Institute [grant number SRG 2014-15].

## References

[CIT0001] Adams JE 2009 Quantitative computed tomography. Eur J Radiol. 71:415–424. 19682815 10.1016/j.ejrad.2009.04.074

[CIT0002] Bochud N, Minonzio JG, Vallet Q, Laugier P 2015 An anisotropic bilayer model to gain insight into *in-vivo* guided wave measurements. Presented at 6th European Symposium on Ultrasonic Characterization of Bone. IEEE; Corfu, Greece 1–4.

[CIT0003] Bossy E, Talmant M, Laugier P 2004 Three-dimensional simulations of ultrasonic axial transmission velocity measurement on cortical bone models. J Acoust Soc Am. 115:2314–2324. 10.1121/1.1689960 15139643

[CIT0004] Castaings M, Lowe M 2008 Finite element model for waves guided along solid systems of arbitrary section coupled to infinite solid media. J Acoust Soc Am. 123:696–708. 10.1121/1.2821973 18247874

[CIT0005] Chen J, Su Z 2014 On ultrasound waves guided by bones with coupled soft tissues: a mechanism study and *in vitro* calibration. Ultrasonics. 54:1186–1196. 10.1016/j.ultras.2013.08.002 24008173

[CIT0006] Chen J, Foiret J, Minonzio JG, Talmant M, Su Z, Cheng L, Laugier P 2012 Measurement of guided mode wavenumbers in soft tissue–bone mimicking phantoms using ultrasonic axial transmission. Phys Med Biol. 57:3025 10.1088/0031-9155/57/10/3025 22538382

[CIT0007] Cheung AM, Adachi JD, Hanley DA, Kendler DL, Davison KS, Josse R, Brown JP, Ste-Marie LG, Kremer R, Erlandson MC, Dian L 2013 High-resolution peripheral quantitative computed tomography for the assessment of bone strength and structure: a review by the canadian bone strength working group. Curr Osteo Rep. 11:136–146. 10.1007/s11914-013-0140-9 PMC3641288 23525967

[CIT0008] COMSOL Users Guide, version 3.2a by COMSOL MULTIPHYSICS; web reference: http://www.comsol.com (Last viewed, 02 May 2015).

[CIT0009] Cooper DM, Matyas JR, Katzenberg MA, Hallgrimsson B 2004 Comparison of microcomputed tomographic and microradiographic measurements of cortical bone porosity. Calcif Tissue Int. 74:437–447. 10.1007/s00223-003-0071-z 14961208

[CIT0010] European Community 1998 Report on osteoporosis in the European community. Strasbourg: EC.

[CIT0011] Fan Z 2010 Applications of guided wave propagation on waveguides with irregular cross-section [PhD dissertation]. London: Imperial College London.

[CIT0012] Foiret J, Minonzio JG, Chappard C, Talmant M, Laugier P 2014 Combined estimation of thickness and velocities using ultrasound guided waves: a pioneering study on *in vitro* cortical bone samples. IEEE Trans Ultrason Ferroelectr Freq Control. 61:1478–1488. 10.1109/TUFFC.2014.3062 25167148

[CIT0013] Grimal Q, Grondin J, Guérard S, Barkmann R, Engelke K, Glüer CC, Laugier P 2013 Quantitative ultrasound of cortical bone in the femoral neck predicts femur strength: results of a pilot study. J Bone Min Res. 28:302–312. 10.1002/jbmr.1742 22915370

[CIT0014] Gurkan UA, Akkus O 2008 The mechanical environment of bone marrow: a review. Ann Biomed Eng. 36:1978–1991. 10.1007/s10439-008-9577-x 18855142

[CIT0015] Haïat G, Naili S, Vu MB, Desceliers C, Soize C 2011 Equivalent contributing depth investigated by a lateral wave with axial transmission in viscoelastic cortical bone. J Acoust Soc Am. 129:EL114–EL120. 10.1121/1.3554719 21476617

[CIT0016] Kanis JA 1994 Assessment of fracture risk and its application to screening for postmenopausal osteoporosis: synopsis of a WHO report. Osteoporos Int. 4:368–381. 10.1007/BF01622200 7696835

[CIT0017] Kanis JA, Melton L3, Christiansen C, Johnston CC, Khaltaev N 1994 The diagnosis of osteoporosis. J Bone Miner Res. 9:1137–1141. 7976495 10.1002/jbmr.5650090802

[CIT0018] Kwun H, Bartels KA, Dynes C 1999 Dispersion of longitudinal waves propagating in liquid-filled cylindrical shells. J Acoust Soc Am. 105:2601–2611. 10.1121/1.426876

[CIT0019] Laugier P, Haïat G 2011 Bone Quantitative Ultrasound. Dordrecht: Springer Vol. 576, Chapters 6–13 10.1007/978-94-007-0017-8

[CIT0020] Lee KI, Yoon SW 2012 Correlations between ultrasonic guided wave velocities and bone properties in bovine tibia *in vitro*. J Acoust Soc Am. 131:EL375–EL381. 10.1121/1.3699532 22559455

[CIT0021] Manogharan P, Rajagopal P, Balasubramaniam K 2016 Longitudinal guided waves confined in radius filler regions of composite joints. J Acoust Soc Am. 2016 Jul 1; 140:334–343. 10.1121/1.4955288 27475157

[CIT0022] Minati NK 2014 Impacts of aquatic taiji exercises on bone mineral density for postmenopausal women. Sci Movement Health. 14:510–515.

[CIT0023] Moilanen P, Nicholson PH, Kilappa V, Cheng S, Timonen J 2007 Assessment of the cortical bone thickness using ultrasonic guided waves: modelling and *in vitro* study. Ultrasound Med Biol. 33:254–262. 10.1016/j.ultrasmedbio.2006.07.038 17306696

[CIT0024] Moilanen P, Talmant M, Kilappa V, Nicholson P, Cheng S, Timonen J, Laugier P 2008 Modeling the impact of soft tissue on axial transmission measurements of ultrasonic guided waves in human radius. J Acoust Soc Am. 124:2364–2373. 10.1121/1.2973228 19062874

[CIT0025] Naili S, Vu MB, Grimal Q, Talmant M, Desceliers C, Soize C, Haïat G 2010 Influence of viscoelastic and viscous absorption on ultrasonic wave propagation in cortical bone: application to axial transmission. J Acoust Soc Am. 127:2622–2634. 10.1121/1.3353091 20370043

[CIT0026] Nguyen VH, Naili S 2012 Simulation of ultrasonic wave propagation in anisotropic poroelastic bone plate using hybrid spectral/finite element method. Int J Numer Method Biomed Eng. 28:861–876. 10.1002/cnm.v28.8 25099567

[CIT0027] Nguyen VH, Naili S 2013 Ultrasonic wave propagation in viscoelastic cortical bone plate coupled with fluids: a spectral finite element study. Comput Methods Biomech Biomed Eng. 16:963–974. 10.1080/10255842.2011.645811 22288934

[CIT0028] Nicholson PH, Moilanen P, Kärkkäinen T, Timonen J, Cheng S 2002 Guided ultrasonic waves in long bones: modelling, experiment and *in vivo* application. Physiol Meas. 23:755 10.1088/0967-3334/23/4/313 12450274

[CIT0029] Niethammer M, Jacobs LJ, Qu J, Jarzynski J 2001 Time-frequency representations of Lamb waves. J Acoust Soc Am. 109:1841–1847. 10.1121/1.1357813 11386539

[CIT0030] Nishiyama KK, Macdonald HM, Buie HR, Hanley DA, Boyd SK 2010 Postmenopausal women with osteopenia have higher cortical porosity and thinner cortices at the distal radius and tibia than women with normal aBMD: an *in vivo* HR-pQCT study. J Bone Miner Res. 25:882–890. 19839766 10.1359/jbmr.091020

[CIT0031] Pattanayak RK, Manogharan P, Balasubramaniam K, Rajagopal P 2015 Low frequency axisymmetric longitudinal guided waves in eccentric annular cylinders. J Acoust Soc Am. 137:3253–3262. 10.1121/1.4921269 26093415

[CIT0032] Predoi MV, Castaings M, Hosten B, Bacon C 2007 Wave propagation along transversely periodic structures. J Acoust Soc Am. 121:1935–1944. 10.1121/1.2534256 17471709

[CIT0033] Protopappas VC, Fotiadis DI, Malizos KN 2006 Guided ultrasound wave propagation in intact and healing long bones. Ultrasound Med Biol. 32:693–708. 10.1016/j.ultrasmedbio.2006.02.001 16677929

[CIT0034] Ramdhas A, Pattanayak RK, Balasubramaniam K, Rajagopal P 2015 Symmetric low-frequency feature-guided ultrasonic waves in thin plates with transverse bends. Ultrasonics. 56:232–242. 10.1016/j.ultras.2014.07.014 25220805

[CIT0035] Ta DA, Huang K, Wang WQ, Wang YY, Le LH 2006 Identification and analysis of multimode guided waves in tibia cortical bone. Ultrasonics. 44:279–284. 10.1016/j.ultras.2006.06.013 16846626

[CIT0036] Ta D, Wang W, Wang Y, Le LH, Zhou Y 2009 Measurement of the dispersion and attenuation of cylindrical ultrasonic guided waves in long bone. Ultrasound Med Biol. 35:641–652. 10.1016/j.ultrasmedbio.2008.10.007 19153000

[CIT0037] Tatarinov A, Sarvazyan N, Sarvazyan A 2005 Use of multiple acoustic wave modes for assessment of long bones: model study. Ultrasonics. 43:672–680. 10.1016/j.ultras.2005.03.004 15982472 PMC2812053

[CIT0038] Thakare DR, Rajagopal P, Belanger P 2016 Ultrasonic guided waves in bone system with degradation. Proceedings of Meetings on Acoust. Acoust Soc Am. 25:020002 10.1121/2.0000147

[CIT0039] Wilcox PD, Lowe MJ, Cawley P 2001 Mode and transducer selection for long range lamb wave inspection. J Intell Mater Syst Struct. 12:553–565. 10.1177/10453890122145348

[CIT0040] Wydra A, Maev RG 2013 A novel composite material specifically developed for ultrasound bone phantoms: cortical, trabecular and skull. Phys Med Biol. 58:N303 10.1088/0031-9155/58/22/N303 24171934

